# T-cell receptor phenotype pattern in atopic children using commercial fluorescently labeled antibodies against 21 human class-specific v segments for the tcrβ chain (vβ) of peripheral blood: a cross sectional study

**DOI:** 10.1186/s13223-016-0115-3

**Published:** 2016-03-02

**Authors:** Gassem Gohal, Christine McCusker, Bruce Mazer, Reza Alizadehfar, Duncan Lejtenyi, Moshe Ben-shoshan

**Affiliations:** Division of Allergy and Clinical Immunology, Department of Pediatrics, Montreal Children’s Hospital, McGill University, 1001 Boulevard Décarie, Room A 02.2227, Montréal, QC H4A 3J1 Canada; McGill University Health Center, 1001 Decarie Blvd Room EM3-2232, Montreal, QC H4A 3J1 Canada

## Abstract

**Background:**

T-cell receptor (TCR) repertoire development is an integral part of the adaptive immune response. T-cell activation requires recognition of appropriately processed antigens by the TCR. Development of a diverse repertoire of TCRs is therefore essential to ensure adequate protection from potential threats. The majority of T-cells in peripheral blood have TCRs composed of an alpha and a beta chain. At the DNA level, the TCR genes are formed through directed recombination from germline sequences—the so-called VDJ recombination [variable (V) joining (J) diversity (D) gene segments] which results in variations in the repertoire. The most variable part of TCRs is the Vβ region (VβTCR), which has multiple V segment families that can be quantitatively measured. However, only sparse data exists on the normal levels of the VβTCR repertoire in healthy children. We aimed to establish normal values for the VβTCR repertoire in atopic children without immunodeficiency.

**Methods:**

Fifty-three children were recruited from food allergy, drug allergy, chronic urticaria and anaphylaxis registries and were divided into groups based on age: >0–2 years, 3–6 years, and 6–18 years. We used commercially available and fluorescently labeled antibodies against 21 human class-specific V segments of the TCRβ chain (Vβ) to study in peripheral blood the quantitative pattern of Vβ variation by flow cytometry.

**Results:**

Children of all ages exhibited a similar pattern of TCR Vβ expression. Vβ 2 was the most commonly expressed family in all three age groups [9.5 % (95 % CI, 8.9, 10 %), 8.8 % (95 % CI, 7.4, 10.2 %) and 7.6 % (7.0, 8.3 %) respectively]. However, the percentage of Vβ 2 decreased in older children and the percentage of Vβ 1 was higher in males. TCR Vβ expression in our sample of atopic children did not differ substantially from previously published levels in non-atopic cohorts.

**Conclusion:**

TCR Vβ diversity follows a predictable and comparable pattern in atopic and healthy non-atopic children. Establishing normal levels for healthy children with and without atopy will contribute to a better definition of Vβ receptor deviation in children with primary immunodeficiency and/or immunodysregulation conditions.

## Background

T-cells play the major effector role in adaptive immune defence [1]. The ability of T-cells to recognise a large variety of antigens is well understood. Since the discovery of the genetic background of the T-cell receptor (TCR), it is now well known that the diversity and specificity of T-cells are a result of gene segment recombination.

There are four distinct T-cell antigen receptor polypeptides (α, β, γ, and δ), which form two different heterodimeric chains (α:β and γ:δ). This results in two subsets of T-cells (T-cell αβ and T-cell γδ) [2]. The majority of T-cells express α:β, while a small percentage express γ:δ [3]. The basic structure of the TCR consists of variable and constant regions, with each region composed of one α and one β chain. The most variable part of the TCR is the variable region of the β chain (Vβ).

The generation of T-cell diversity occurs during the assembly of the TCR in the thymus through the genetic recombination of the V, D, and J segments [variable (V) joining (J) diversity (D) gene segments] [4]. The β chain is generated by the VDJ recombination, whereas the alpha chain is generated by the VJ recombination. This process is believed to occur through a random recombination of gene segments, and it produces a diverse repertoire of Vβ (VβTCR). In later steps, before the full maturation of T-cells and before the cells leave the thymus; the TCR has to appropriately bind a self MHC antigen (positive selection) [5]. The T-cells with TCRs that inappropriately bind self MHC antigens die by apoptosis.

The assessment of the Vβ repertoire of TCRs has classically been performed using two different methodologies. Complementarity determining regions (CDRs) length spectratyping is a genetic assay that uses the polymerase chain reaction (PCR). This method provides qualitative information about TCR Vβ clonality. Another method involves flow cytometry analyses of TCR Vβ families labeled with specific monoclonal antibodies. This method provides a quantitative assessment of TCR Vβ clones and is well established in clinical settings. The latter method provides a faster generation of results and is relatively easier and less expensive than the former. In addition, it has a high degree of reproducibility. The development of a large panel of monoclonal antibodies to TCRs, mainly against Vβ epitopes, has permitted the study of the TCR repertoire. By using commercial fluorescently labeled antibodies that cover over 75 % of the whole TCR Vβ repertoire, it is possible to quantify the cells that express a different Vβ repertoire. These allow for early diagnosis and follow up as well as response to medical management of primary immunodeficiency diseases, immunodysregulation disorders, and malignancies [6–8].

There is a limited amount of data on the normal levels of the VβTCR repertoire in healthy subjects, and only a few studies on healthy children exist [9–11]. Further, although there is an association between certain immunodeficiencies and atopy, there are currently no studies assessing the potential effect of atopy on the VβTCR repertoire [12]. We aimed to determine the VβTCR repertoire in atopic children without immunodeficiencies, to compare the results to non-atopic children and to assess the effects of age, sex and different atopic co-morbidities.

## Methods

The study was conducted between January 2014 and February 2015 in Montreal, Canada. Subjects were recruited from the allergy and immunology clinic of the Montreal Children’s Hospital. The study population included 53 healthy atopic Caucasian children under the age of 18. We excluded subjects with active or recent infections, those taking immunosuppressive medications, and those with any known chronic medical conditions other than atopy. The subjects were divided into groups based on age: >0–2 years, 3–6 years, and 7–18 years. Whole blood samples were taken from the children during their clinical evaluation.

Vβ TCR analysis was performed using commercially available fluorescently labelled antibodies against the following 21 human class-specific V segments of the TCRβ chain: Vβ 1, Vβ 2, Vβ 3, Vβ 4, Vβ 5.2, Vβ 5.3, Vβ 7.1, Vβ 7.2, Vβ 8, Vβ 9, Vβ 11, Vβ 12, Vβ 13.1, Vβ 13.2, Vβ 13.6, Vβ 14, Vβ 16, Vβ 17, Vβ 21.3, Vβ 22 and Vβ 23.

Vβ staining was determined using modified three-color flow cytometry with the IOTest Beta Mark TCR Repertoire Kit (Beckman Coulter, Marseille, France), which consists of monoclonal antibodies (mAbs) designed to identify 21 distinct TCR Vβ families. Each set consisted of three distinct anti-Vβ family-specific mAb labelled with fluorescein isothiocyanate (FITC), phycoerythrin (PE) or doubly labelled with FITC and PE. Fresh whole blood was stained simultaneously with phycoerythrin-cyanine5 conjugated anti-CD3 (clone UCHT1). Hundred microlitre of whole blood was washed prior to incubation with phycoerythrin-cyanine5 conjugated anti-CD3 antibody with 20 μl of appropriate TCR-Vβ antibody for 30 min in the dark at room temperature. Erythrocytes were lysed using fluorescence activated cell sorter (FACS) lysing solution (Becton–Dickinson, Oxford, UK) and cells were then washed. At least 30,000 CD3+ lymphocyte events were collected for analysis. The CD3+, lymphocytes were gated using forward- and side-scatter characteristics and cell populations gated according to CD3 expression.

**Fig. 1:**
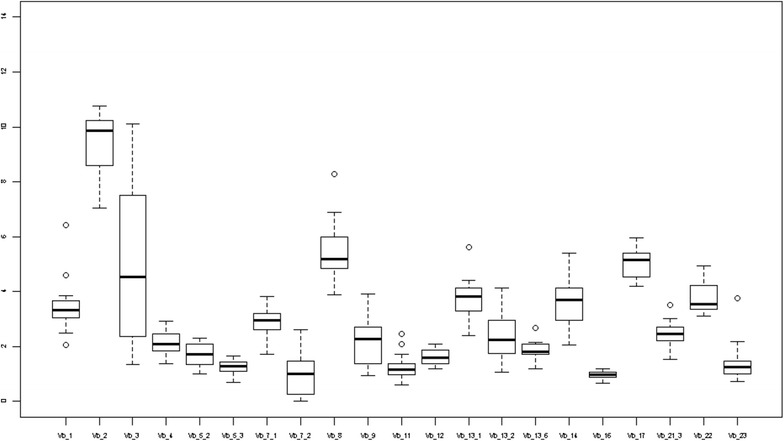
Box plots for VβTCR percentages of all CD3 cells in 53 children with atopy

Data acquisition was performed using a FACS Calibur flow cytometer and Cellquest software (BD Biosciences) (Fig. 1).

The results of the data were expressed as mean ± 95 % confidence interval (CI). Uni- and multivariate linear regressions were used to control for potential confounders and assess the effects of age, sex, and atopy on VβTCR. All statistical analyses were conducted using R version 2.12.0 (2010-10-15). This study received ethics approval from the McGill Research Ethics Board.

## Results

Among the 53 children assessed, 53 % were males. The majority had food allergies and chronic urticaria (Table 1).

**Table 1:** Demosgraphic and atopic characteristics

Variable	% (95 % CI)
Age (years, median, IQR: interquartile range)	6 (2, 13)
Males (%)	52.8 (38.8, 66.5)
Atopic condition	
Food allergy	49.1 (35.2, 63.0)
Chronic urticaria	35.8 (23.5, 50.2)
Hay fever	7.5 (2.4, 19.1)
Asthma	1.9 (0.1, 11.4)
Drug allergy	5.7 (1.5, 16.6)

**Fig. 2:**
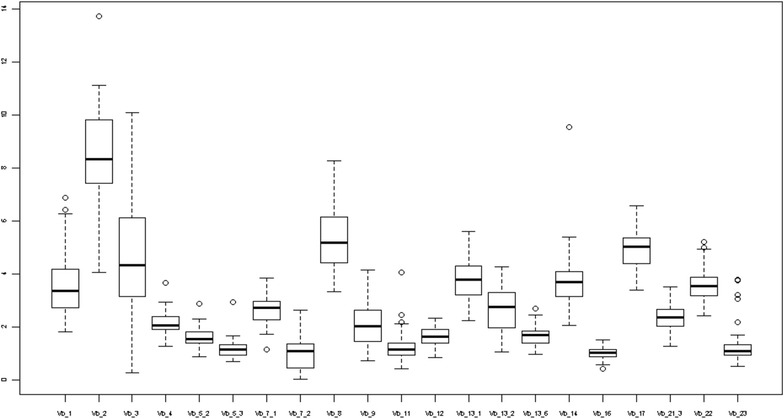
Box plots for VβTCR percentages of all CD3 cells for children 0–2 years old

Our results reveal that Vβ 2 composed the majority of VβTCRs in our sample (Fig. 2). The next most highly expressed members of the Vβ repertoire were Vβ 8 and Vβ 17. An important observation in this study was that the pattern of VβTCRs for atopic children in different pediatric age groups followed a pattern similar to values previously reported in non-atopic children (Table 2) [10].

**Table 2:** Comparison of the means of VβTCR expression in a topic cohort with healthy control from McLean-Tooke et al. study

	0–2 years n = 16 Gassem g et al.	0–9 months n = 5 McLean-Tooke et al.	3–6 years n = 12 Gassem g et al.	2–5 years n = 10 McLean-Tooke et al.	7–18 n = 25 Gassem g et al.	10–16 n = 10 McLean-Tooke et al.
Vβ_1	*3.5*	*3.6*	3.6	3·9	*3.6*	*4.0*
Vβ_2	*9.5*	*9.9*	8.8	8·8	*7.6*	*8.7*
Vβ_3	*4.9*	*5.7*	4.1	4·9	*3.5*	*3.7*
Vβ_4	*2.1*	*2.1*	2.0	2·1	*2.2*	*2.2*
Vβ_5.2	*1.7*	*1.3*	1.7	1·7	*2.2*	*1.6*
Vβ_5.3	*1.3*	*0.73*	1.3	1·2	*1.1*	*1.0*
Vβ_7.1	*2.9*	*2.7*	2.5	2·6	*2.6*	*2.7*
Vβ_7.2	*1.0*	*1.0*	1.2	1·5	*1.0*	*1.6*
Vβ_8	*5.4*	*4.5*	5.4	5·4	*5.2*	*5.2*
Vβ_9	*2.2*	*2.9*	1.8	3·3	*2.1*	*3.4*
Vβ_11	*1.3*	*0.9*	1.1	1·0	*1.3*	*1.0*
Vβ_12	*1.6*	*1.6*	1.7	1·8	*1.6*	*1.7*
Vβ_13_1	*3.7*	*4.5*	4.3	5·0	*3.6*	*4.5*
Vβ_13_2	*2.4*	*3.1*	2.9	3·5	*2.8*	*4.0*
Vβ_13.6	*1.8*	*1.8*	1.6	1·8	*1.5*	*1.8*
Vβ_14	*3.6*	*4.5*	3.5	4·5	*4.0*	*4.0*
Vβ_16	*1.0*	*1.2*	1.0	0·9	*1.0*	*1.1*
Vβ_17	*5.0*	*5.1*	4.7	5·7	*5.0*	*5.4*
Vβ_21.3	*2.5*	*2.5*	2.3	2·5	*2.3*	*2.5*
Vβ_22	*3.8*	*3.0*	3.4	3·57	*3.6*	*3.6*
Vβ_23	*1.4*	*0.7*	1.4	1·1	*1.1*	*1.1*

**Fig. 3:**
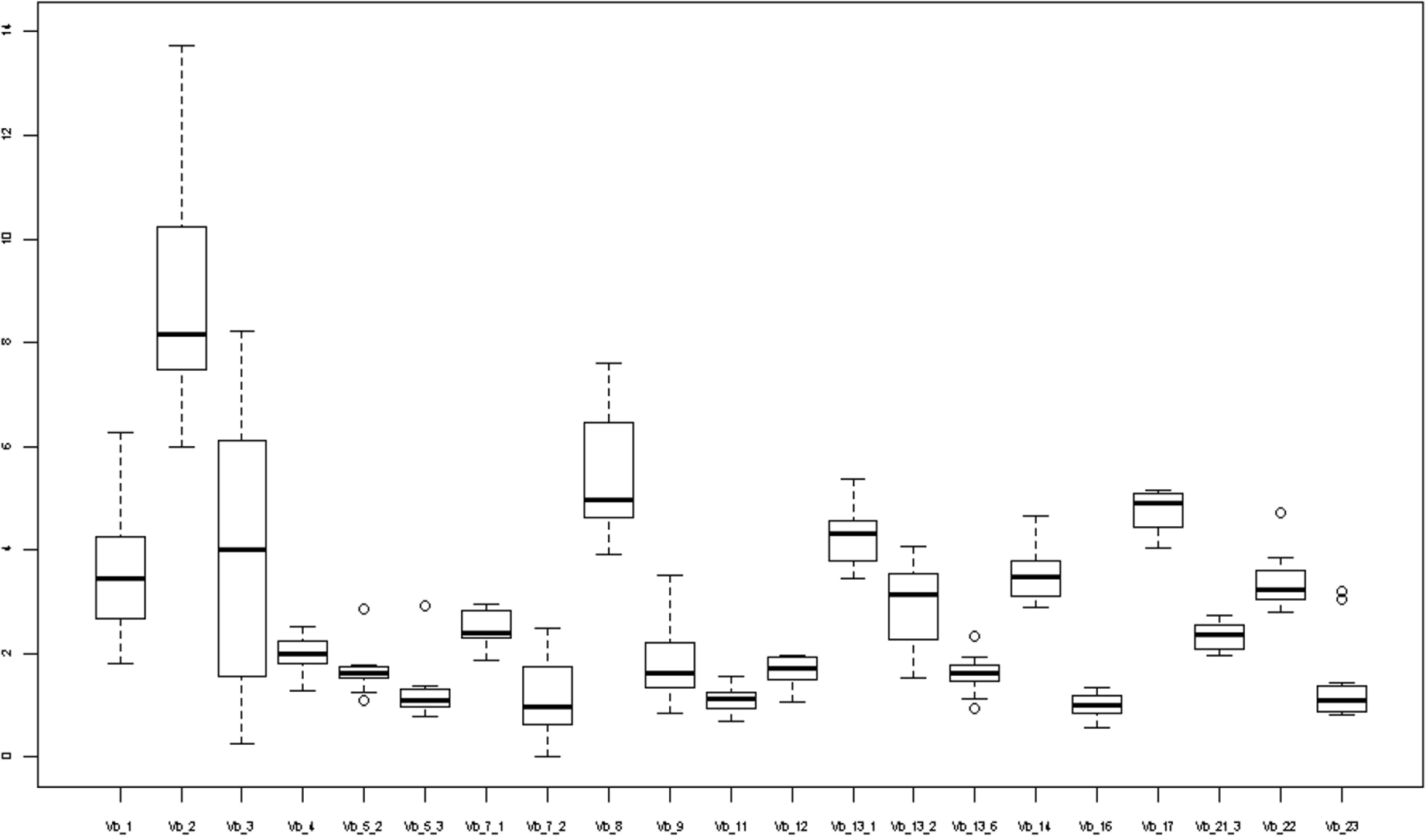
Box plots for VβTCR percentages of all CD3 cells for children 3–6 years old

**Fig. 4:**
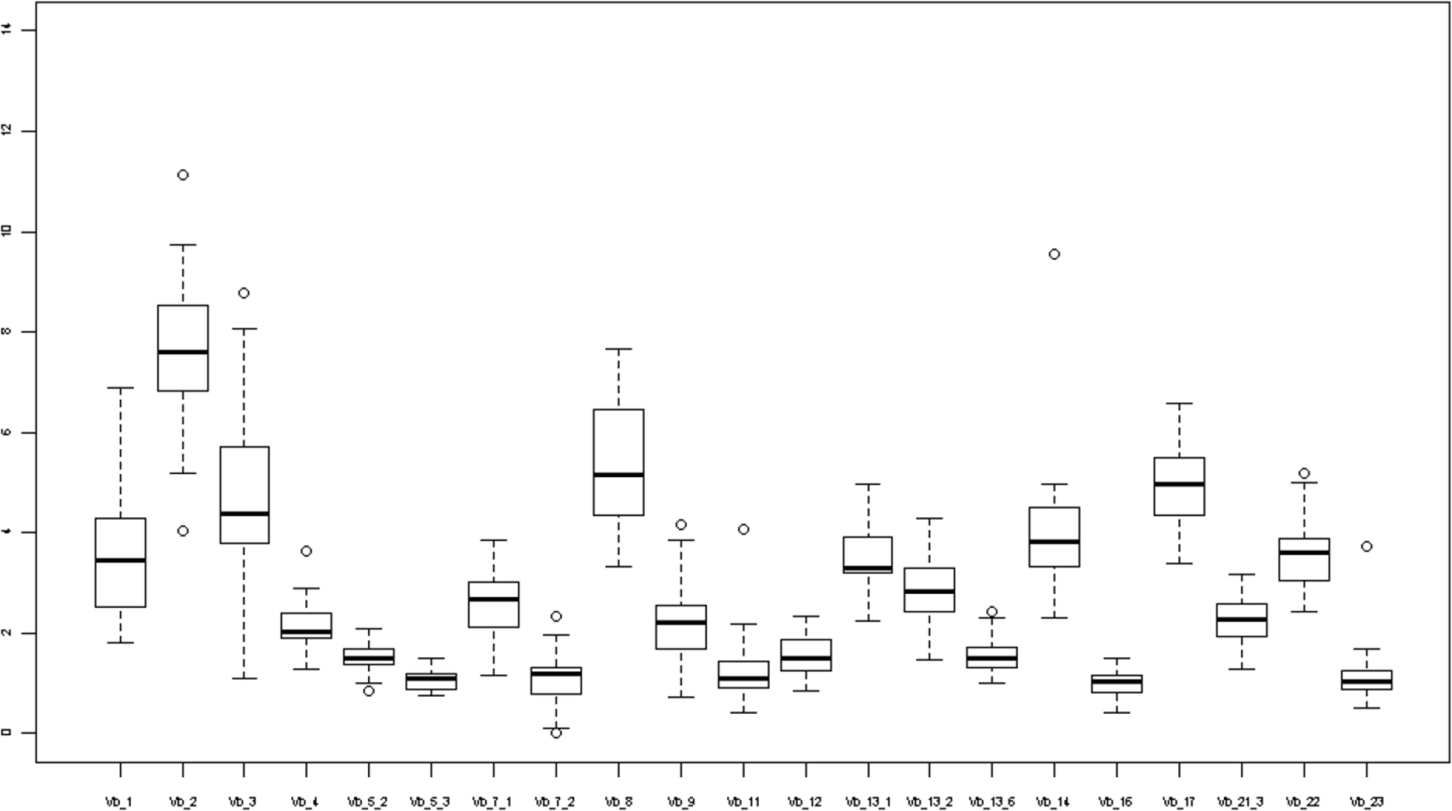
Box plots for VβTCR percentages of all CD3 cells for children 7–18 years old

Comparing uni and multivariate logistic regressions revealed that age and sex affected the levels of Vβ 2 and Vβ 1 respectively. The levels of Vβ 2 decreased in older children [beta adjusted for sex and atopic condition = −0.1, (95 % CI, −0.2 −0.03)] and males had higher levels of Vβ 1 [beta adjusted for age and atopic condition = 0.7, 95 % CI (0.1 1.3)]. Given the effects of age and sex, we assessed VβTCRs for three age groups: 0–2 years old, 3–6 years old, and 7–18 years old for males and females (Figs. 2, 3 and 4 and Tables 3, 4 and 5). We did not find significant associations between the type of atopic condition and the level of a specific Vβ chain.

**Table 3:** VβTCR percentages for children 0–2 years

	% Mean (95 % CI) All (N = 12)	% Mean (95 % CI) Male (N = 5)	% Mean (95 % CI) Female (N = 7)
Age years (IQR)	4.4 (3.0, 5.2)	5.0 (3.0, 5.0)	4.0 (3.5, 5.5)
Sex (% males)	41.7 % (16.5, 71.4)		
Vβ_1	3.6 (2.8, 4.4)	4.1 (2.4, 5.8)	3.2 (2.2, 4.2)
Vβ_2	8.8 (7.4, 10.2)	8.2 (6.1, 10.4)	9.2 (6.8, 11.5)
Vβ_3	4.1 (2.4, 5.8)	5.6 (2.1, 9.1)	3.0 (1.1, 4.9)
Vβ_4	2.0 (1.8, 2.2)	2.0 (1.7, 2.3)	2.0 (1.6, 2.4)
Vβ_5.2	1.7 (1.4, 1.9)	1.7 (0.8, 2.6)	1.6 (1.5, 1.7)
Vβ_5.3	1.3 (0.9, 1.6)	1.4 (0.4, 2.5)	1.1 (0.9, 1.3)
Vβ_7.1	2.5 (2.3, 2.7)	2.5 (1.9, 3.0)	2.5 (2.3, 2.8)
Vβ_7.2	1.2 (0.6, 1.7)	1.7 (0.6, 2.8)	0.8 (0.3, 1.2)
Vβ_8	5.4 (4.7, 6.2)	5.3 (4.2, 6.5)	5.5 (5.0, 6.0)
Vβ_9	1.8 (1.4, 2.3)	2.1 (0.9, 3.2)	1.7 (1.1, 2.2)
Vβ_11	1.1 (1.0, 1.3)	1.2 (0.9, 1.6)	1.0 (0.8, 1.2)
Vβ_12	1.7 (1.5, 1.8)	1.8 (1.5, 2.1)	1.6 (1.3, 1,8)
Vβ_13_1	4.3 (3.9, 4.7)	4.6 (3.7, 5.6)	4.1 (3.7, 4.4)
Vβ_13_2	2.9 (2.4, 3.4)	3.3 (2.4, 4.2)	2.6 (1.8, 3.3)
Vβ_13.6	1.6 (1.4, 1.8)	1.6 (1.2, 1.9)	1.6 (1.2, 2.0)
Vβ_14	3.5 (3.2, 3.8)	3.6 (3.1, 4.0)	3.5 (2.9, 4.1)
Vβ _16	1.0 (0.8, 1.1)	1.0 (0.6, 1.4)	1.0 (0.8, 1.1)
Vβ_17	4.7 (4.5, 5.0)	4.6 (3.9, 5.3)	4.8 (4.5, 5.1)
Vβ_21.3	2.3 (2.2, 2.5)	2.4 (2.1, 2.8)	2.3 (2.0, 2.5)
Vβ_22	3.4 (3.1, 3.7)	3.1 (2.9, 3.3)	3.6 (3.1, 4.2)
Vβ_23	1.4 (0.9, 1.9)	1.9 (0.6, 3.3)	1.0 (0.8, 1.2)

**Table 4:** VβTCR percentages for children 3–6 years

	% Mean (95 % CI) All (N = 16)	% Mean (95 % CI) Male (N = 11)	% Mean (95 % CI) Female (N = 5)
Age years (IQR)	1.0 (1.0, 2.0)	1.0 (0.9, 2)	1.0 (1.0, 1.0)
Sex (% males)	68.8 (41.5, 87.9)		
Vβ_1	3.5 (2.9, 4.0)	3.6 (2.8, 4.4)	3.2 (2.7, 3.8)
Vβ_2	9.5 (8.9, 10.0)	9.3 (8.5, 10.1)	9.8 (8.7, 11)
Vβ_3	4.9 (3.4, 6.5)	5.2 (3.2, 7.1)	4.5 (0.3, 8.7)
Vβ_4	2.1 (0.5, 3.7)	2.1 (1.8, 2.4)	2.2 (1.8, 2.6)
Vβ_5.2	1.7 (1.5, 1.9)	2.1 (1.8, 2.3)	2.0 (1.5, 2.4)
Vβ_5.3	1.3 (1.1, 1.4)	1.3 (1.0, 1.5)	1.3 (1.1, 1.4)
Vβ_7.1	2.9 (2.6, 3.2)	2.9 (2.7, 3.2)	2.7 (1.6, 3.9)
Vβ_7.2	1.0 (0.6, 1.4)	1.1 (0.5, 1.6)	1.0 (0.3, 1.7)
Vβ_8	5.4 (5.0, 5.8)	5.1 (4.5, 5.7)	6.1 (5.4, 6.8)
Vβ_9	2.2 (1.7, 2.6)	1.9 (1.3, 2.4)	2.8 (2.0, 3.7)
Vβ_11	1.3 (1.0, 1.5)	1.2 (0.5, 1.6)	2.0 (1.5, 2.4)
Vβ_12	1.6 (1.5, 1.8)	1.6 (1.4, 1.9)	1.5 (1.2, 1.9)
Vβ_13_1	3.7 (3.3, 4.2)	4.1 (3.7, 4.5)	2.9 (2.2, 3.6)
Vβ_13_2	2.4 (1.9, 2.9)	2.6 (1.6, 2.2)	2.9 (1.8, 3.9)
Vβ_13.6	1.8 (1.6, 2.0)	1.8 (1.5, 2.0)	1.9 (1.4, 2.5)
Vβ_14	3.6 (3.2, 4.1)	3.7 (3.2, 4.3)	3.4 (2.2,4.6)
Vβ_16	1.0 (0.9, 1.0)	1.0 (0.9, 1.1)	1.0 (0.8, 1.1)
Vβ_17	5.0 (4.7, 5.3)	5.0 (4.7, 5.4)	5.0 (4.2, 5.8)
Vβ_21.3	2.5 (2.2, 2.7)	2.7 (2.1, 2.7)	2.5 (1.8, 3.2)
Vβ_22	3.8 (3.5, 4.1)	3.8 (3.4, 4.2)	3.7 (3.2, 4.2)
Vβ_23	1.4 (1.0, 1.8)	1.4 (0.8, 2.0)	1.4 (0.9, 2.0)

**Table 5:** VβTCR percentages for children 7–18 years old

	% Mean (95 % CI) All (N = 25)	% Mean (95 % CI) Male (N = 15)	% Mean (95 % CI) Female (N = 13)
Age months (IQR)	13.0 (8.0, 16.0)	12.5 (10.3, 15.0)	14.0 (8.0, 16.0)
Sex (% males)	48.0 (28.3, 68.2)		
Vβ_1	3.6 (3.1, 4.0)	4.0 (3.2, 4.9)	3.1 (2.5, 3.6)
Vβ_2	7.6 (7.0, 8.3)	7.3 (6.4, 8.1)	8.0 (7.0, 9.1)
Vβ_3	4.5 (3.6, 5.3)	4.9 (3.8, 5.9)	4.4 (3.0, 5.8)
Vβ_4	2.2 (2.2, 2.4)	2.3 (1.9, 2.6)	2.1 (1.9, 2.3)
Vβ_5.2	2.2 (2.0, 2.3)	1.6 (1.3, 1.8)	1.5 (1.3, 1.7)
Vβ_5.3	1.1 (1.0, 1.2)	1.1 (1.0, 1.3)	1.0 (0.9, 1.1)
Vβ_7.1	2.6 (2.3, 2.9)	2.6 (2.0, 3.1)	2.6 (2.3, 3.0)
Vβ_7.2	1.0 (0.8, 1.2)	1.0 (0.8, 1.3)	1.0 (0.5, 1.4)
Vβ_8	5.2 (5.0, 5.5)	5.4 (5.2, 5.6)	5.1 (4.6, 5.5)
Vβ_9	2.1 (1.8, 2.5)	2.2 (1.6, 2.9)	2.0 (1.7, 2.4)
Vβ_11	1.3 (1.0, 1.6)	1.2 (0.9, 1.4)	1.4 (0.8, 1.9)
Vβ_12	1.6 (1.4, 1.8)	1.6 (1.3, 1.8)	1.6 (1.4, 1.9)
Vβ_13_1	3.6 (3.3, 3.9)	3.6 (3.2, 4.0)	3.6 (3.2, 4.1)
Vβ_13_2	2.8 (2.5, 3.1)	2.9 (2.5, 3.4)	2.7 (2.2, 3.1)
Vβ_13.6	1.5 (1.4, 1.7)	1.6 (1.3, 1.8)	1.5 (1.3, 1.7)
Vβ_14	4.0 (3.4, 4.5)	4.1 (2.9, 5.2)	3.9 (3.4, 4.3)
Vβ_16	1.0 (0.9, 1.1)	0.9 (0.8, 1.0)	1.1 (0.9, 1.2)
Vb_17	5.0 (4.6, 5.3)	5.0 (4.9, 5.4)	5.0 (4.4, 5.6)
Vβ_21.3	2.3 (2.1, 2.5)	2.3 (2.0, 2.6)	2.2 (1.9, 2.5)
Vβ_22	3.6 (3.3, 3.9)	3.7 (3.1, 4.2)	3.5 (3.2, 3.9)
Vβ_23	1.1 (0.9, 1.4)	1.2 (0.7, 1.8)	1.0 (0.8, 1.3)

## Discussion

We report here the VβTCR repertoire in the largest sample to date of children with no immunodeficiency. Further, our study is the first to assess the VβTCR repertoire in children with atopy. Our results reveal a similar distribution of VβTCRs in atopic versus previously reported non-atopic children. In addition, we have found that age and sex may affect the VβTCR repertoire.

There are several studies of the Vβ repertoire aimed at identifying the clonal pattern of TCRs or determining specific Vβ region(s) in different pathological conditions where T-cells have a fundamental role [10] for example: autoimmune disorders [13, 14] HIV infection [15], malignancy [16], asthma [17], and immunodeficiency [18, 19]. VβTCR assays have also been used in disease evaluation and for monitoring the progression and response of treatments [17, 20, 21]. These studies have usually compared their results with a small sample of healthy controls [10].

We analysed 21 different VβTCR families in CD3+ T lymphocytes. Our estimates were similar to previously published estimates in children [10]. Hence, we deduce that atopy does not affect substantially the levels of VβTCR.

Older reports of flow cytometric analyses of TCR repertoire used small numbers of Vβ monoclonal antibodies and therefore give a less accurate picture of the diversity of the normal repertoire. Our study, using 21 monoclonal antibodies against human class-specific V segments of the TCRβ chain (Vβ), covers almost 75 % of the whole repertoire. Further, our study is the first to detect the potential effects of age and sex on VβTCR repertoire likely to our larger sample size.

The study has some potential limitations. Given that no studies have thus far reported on VβTCR repertoire in a large sample of atopic children, it is possible that the effects observed due to age and sex are confounded by the presence of atopy. Large scale studies in non atopic children are required in order to address this potential limitation. However, given that an association between immunodeficiency, immune dysregulation, and atopy is often reported particularly in cases of antibody deficiency [12]. Our estimates are nonetheless useful even if they apply only to comparisons within atopic populations. In addition, the comparison between our study in atopic children and previous studies conducted in non- atopic children might have been affected by differences in study devices and batch of the commercially available mono clonal antibodies. Another potential limitation of our study is that we were not able to exclude patients with asymptomatic chronic viral infections (e.g., cytomegalovirus (CMV), Epstein-Barr virus (EBV), herpes simplex virus (HSV), which might affect the VβTCR repertoire [22]. However, previous studies suggest that asymptomatic infections would not affect substantially the VβTCR [23]. Although previous studies suggest that specific food allergies may effect specific VβTCR chains we did not observe such an association likely due to the relatively small sample size of children with a specific food allergy [24].

## Conclusion

In conclusion, our findings serve to establish normal reference values of VβTCR levels in atopic children and would contribute to detect deviations in this repertoire in atopic children with suspected immunodeficiency and immune-dysregulations and malignancies Future studies assessing such comparisons are required to elucidate disparities that have clinical implications for diagnosis and management.

## Abbreviations

CDR3: complementarity determining regions
CI: confidence interval
CMV: cytomegalovirus
EBV: Epstein-Barr virus
FACS: fluorescence activated cell sorter
FITC: fluorescein isothiocyanate
HSV: herpes simplex virus
IQR: interquartile range
MAbs: monoclonal antibodies
PE: phycoerythrin
TCR: T-cell receptor
VDJ: variable (V), joining (J), diversity (D) gene segments
